# Geographic Location and Corporate Ownership of Hospitals in Relation to Unfilled Positions in the 2023 Emergency Medicine Match

**DOI:** 10.5811/westjem.18436

**Published:** 2024-03-29

**Authors:** Zachary J. Jarou, Angela G. Cai, Leon Adelman, David J. Carlberg, Sara P. Dimeo, Jonathan Fisher, Todd Guth, Bruce M. Lo, Laura Oh, Rahul Puttagunta, Gillian R. Schmitz

**Affiliations:** * Ascension Providence Hospital, Department of Emergency Medicine, Southfield, Michigan; † Michigan State University, East Lansing, Michigan; ‡ University of Pennsylvania, Department of Emergency Medicine, Philadelphia, Pennsylvania; § Ivy Clinicians, Raleigh, North Carolina; ∥ Georgetown University, Department of Emergency Medicine, Washington DC; ¶ Dignity Health East Valley Rehabilitation Hospital, Chandler Regional Medical Center, Chandler, Arizona; # Texas Christian University, Burnett School of Medicine, Fort Worth, Texas; ** University of Colorado School of Medicine, Department of Emergency Medicine, Denver, Colorado; †† Sentara Norfolk General Hospital, Virginia; ‡‡ Eastern Virginia Medical School, Virginia; §§ Emory University, Department of Emergency Medicine, Atlanta, Georgia; ∥∥ Uniformed Services University of Health Sciences, Department of Military and Emergency Medicine, Bethesda, Maryland

## Abstract

**Introduction:**

In the 2023 National Resident Matching Program (NRMP) match, there were 554 unfilled emergency medicine (EM) positions before the Supplemental Offer and Acceptance Program (SOAP). We sought to describe features of EM programs that participated in the match and the association between select program characteristics and unfilled positions.

**Methods:**

The primary outcome measures included the proportion of positions filled in relation to state and population density, hospital ownership type, and physician employment model. Secondary outcome measures included comparing program-specific attributes between filled and unfilled programs, including original accreditation type, year of original accreditation, the total number of approved training positions, length of training, urban-rural designation, hospital size by number of beds, resident-to-bed ratio, and the percentage of disproportionate share patients seen.

**Results:**

The NRMP Match had 276 unique participating EM programs with 554 unfilled positions. Six states offered 52% of the total NRMP positions available. Five states were associated with two-thirds of the unfilled positions. Public hospitals had a statistically significant higher match rate (88%) when compared to non-profit and for-profit hospitals, which had match rates of 80% and 75%, respectively (*P* < 0.001). Programs with faculty employed by a health system had the highest match rate of 87%, followed by clinician partnerships at 79% and private equity groups at 68% (*P* < 0.001 overall and between all subgroups).

**Conclusion:**

The 2023 match in EM saw increased rates in the number of residency positions and programs that did not fill before the SOAP. Public hospitals had higher match rates than for-profit or non-profit hospitals. Residency programs that employed academic faculty through the hospital or health system were associated with higher match rates.

Population Health Research Capsule What do we already know about this issue?

*Prior studies examined program features and ownership predictors of unfilled positions but without deeper analysis of corporate ownership trends and associations.*

What was the research question?

*What program features and hospital or faculty ownership are associated with the unfilled 2023 match?*

What was the major finding of the study?

*Public, for-profit, and non-profit matched 88%, 80%, and 75% (P < 0.001). Program faculty employed, clinician partnership, and private equity matched 87%, 79%, and 68% (P < 0.001).*

How does this improve population health?

*Understanding factors for match success help ensure stable inputs to the emergency medicine workforce.*

## INTRODUCTION

Emergency medicine (EM) has historically been a highly competitive specialty, filling all or nearly all the available residency positions as part of the Main Residency Match (match) organized by the National Residency Matching Program (NRMP). After a record number of applicants in 2021, the past two years have seen a decline in the number of student applicants while the number of available EM residency positions has continued to increase, ultimately resulting in a rise in unfilled programs and positions. In the 2022 NRMP match, there were 219 unfilled EM positions among 69 programs before the Supplemental Offer and Acceptance Program (SOAP), and in 2023 that figure approximately doubled to 554 unfilled positions among 131 programs. Many are concerned that the dramatic increase in pre-SOAP unfilled positions represents a decline in the desirability and competitiveness of the specialty.^1^

This is an observational study describing features of EM residency programs that participated in the 2023 NRMP match and the association between select program characteristics and unfilled positions. It is unclear whether certain characteristics including state-based geographic location and population density, hospital financing models, faculty physician employment models, or specific program characteristics such as the size of program or length of training are associated with higher rates of unfilled positions. Transparency of factors associated with unfilled positions will guide the specialty’s response to the match and program accreditation requirements with objective data. Prior studies have examined similar factors but provided limited detail and nuance on the topic of corporate ownership, which we expound upon in our study.^2^^,^^3^

## METHODS

### Study Design and Setting

In this observational study we used publicly available datasets to analyze the match results for EM residency programs participating in the 2023 NRMP Match based on STROBE guidelines.^4^ The institutional review board determined this study to be exempt. All EM residency programs and the positions they offered that participated in the 2023 NRMP Match were included in the study.

### Variables and Measurements

We obtained a list of EM residency programs and their number of offered and filled positions from the NRMP. Each NRMP ID was linked to the program’s Accreditation Council for Graduate Medical Education (ACGME) Program ID, which provided information about the year of accreditation, program length, number of approved positions, and training sites. We also obtained a list of ACGME programs that were formally accredited by the American Osteopathic Association (AOA) and the year of earliest AOA accreditation type. The ACGME Site ID for each primary site was linked to the hospital’s Centers for Medicare and Medicaid Services (CMS) Certification Number and the 2023 CMS Inpatient Prospective Payment System Final Rule Data, which includes information about hospital ownership type, urban-rural location, number of hospital beds, resident-to-bed ratio, and percentage of disproportionate share hospital (DSH) patients. Hospitals were linked to the health systems that operate them. Information about the physician group staffing each hospital’s emergency department and the ownership type of those groups as of March 2023 was obtained from Ivy Clinicians.^5^ We defined physician groups as “private equity” if there was a majority-ownership interest by a private equity firm. “Clinician partnerships” were defined as being majority owned by physicians. This included independent faculty physician groups affiliated with a health system, equal-partnership democratic groups, groups where certain clinicians may own a larger percentage of shares, and groups with minority-interest ownership by a private equity firm. We defined physician groups as “health system” if they were employed directly by the physician organization of the hospital, health system, medical school, or academic medical center.

### Outcomes Measures

The primary outcome measures included the proportion of positions filled by state and population density, hospital ownership type, and physician employment model. Secondary outcome measures compared other program-specific attributes between filled and unfilled programs, including original accreditation type, year of original accreditation, year of ACGME accreditation, the total number of ACGME-approved training positions, length of training, urban-rural designation, hospital size by number of beds, resident-to-bed ratio, and the percentage of DSH patients seen. A program was classified as unfilled if there were one or more unmatched positions across any of its NRMP IDs; programs with zero unfilled positions across any of its NRMP IDs were classified as filled.

### Statistical Methods

We performed all data extraction, transformation, and analysis using RStudio version 2023.03.0 + 386 running R version 4.2.3 (RStudio, PBC, Boston, MA). We described continuous variables using medians and interquartile ranges. Categorical variables were described using frequency and percentages. We compared continuous variables using the Wilcoxon rank-sum test. We compared categorical variables using Pearson chi-squared testing with Bonferroni post-hoc analysis where more than two groups were compared. *P*-values less than 0.05 were considered to be statistically significant.

## RESULTS

### Characteristics of Study Subjects

As of March 2023, there were 283 ACGME-accredited EM residencies; however, five of these were military programs that do not historically participate in the NRMP match, and there were two additional programs that did not participate in the 2023 match. There were 11 EM programs with dual NRMP IDs, where one of the IDs may be used to offer a single position to a special type of applicant, such as international/private-funded positions, research positions, or for three-year MD path residents.^6^ A total of 276 EM programs participated in the match, offering 3,010 positions in 43 states plus the District of Columbia and Puerto Rico. There were 131 programs (48%) with 554 positions (18%) that were unfilled before the SOAP.

### Geography

Six states offered 52% of the total NRMP EM positions available: New York (338), California (285), Michigan (236), Florida (234), Pennsylvania (234), and Texas (184). There was significant variation in the number of residency positions available per state population. Among the six states that offered the largest number of residency positions, Michigan had the most NRMP positions per population at 23.5 residents per million citizens in the 2020 census, while Texas had only 6.1 residents per million citizens. Five states were associated with two-thirds of the unfilled positions: Michigan (92); New York (83); Pennsylvania (78); Ohio (56); and Florida (49). There was also significant variation in the percentage of unmatched positions by state (Table 1).

**Table 1.** Residency match results by state and emergency medicine positions per state population.

State	Number of programs	NRMP Quota	NRMP unmatched	Percent matched	Percent unmatched	2020 population (millions)	Residents per population (millions)
Alabama	2	18	1	94%	6%	5.1	3.5
Arizona	5	51	5	90%	10%	7.4	6.9
Arkansas	2	16	9	44%	56%	3	5.3
California	24	285	22	92%	8%	39	7.3
Colorado	1	17	0	100%	0%	5.8	2.9
Connecticut	2	37	0	100%	0%	3.6	10.2
Delaware	2	18	6	67%	33%	1	17.7
District of Columbia	2	22	0	100%	0%	0.7	32.7
Florida	22	234	49	79%	21%	22.2	10.5
Georgia	5	58	6	90%	10%	10.9	5.3
Illinois	12	144	9	94%	6%	12.6	11.4
Indiana	1	21	0	100%	0%	6.8	3.1
Iowa	1	10	0	100%	0%	3.2	3.1
Kansas	1	10	4	60%	40%	2.9	3.4
Kentucky	2	25	0	100%	0%	4.5	5.5
Louisiana	4	42	0	100%	0%	4.6	9.1
Maine	1	10	0	100%	0%	1.4	7.2
Maryland	2	23	0	100%	0%	6.2	3.7
Massachusetts	5	72	2	97%	3%	7	10.3
Michigan	25	236	92	61%	39%	10	23.5
Minnesota	3	32	0	100%	0%	5.7	5.6
Mississippi	3	28	9	68%	32%	2.9	9.5
Missouri	5	51	11	78%	22%	6.2	8.3
Nebraska	1	12	0	100%	0%	2	6.1
Nevada	3	25	11	56%	44%	3.2	7.9
New Hampshire	1	6	0	100%	0%	1.4	4.3
New Jersey	12	122	27	78%	22%	9.3	13.2
New Mexico	1	12	0	100%	0%	2.1	5.7
New York	31	388	83	79%	21%	19.7	19.7
North Carolina	7	85	22	74%	26%	10.7	7.9
Ohio	17	158	56	65%	35%	11.8	13.4
Oklahoma	5	33	8	76%	24%	4	8.2
Oregon	1	11	0	100%	0%	4.2	2.6
Pennsylvania	23	234	78	67%	33%	13	18
Puerto Rico	2	16	1	94%	6%	3.2	5
Rhode Island	2	22	3	86%	14%	1.1	20.1
South Carolina	5	55	4	93%	7%	5.3	10.4
Tennessee	5	48	5	90%	10%	7.1	6.8
Texas	15	184	15	92%	8%	30	6.1
Utah	1	12	0	100%	0%	3.4	3.5
Vermont	1	6	0	100%	0%	0.6	9.3
Virginia	6	63	13	79%	21%	8.7	7.3
Washington	1	17	0	100%	0%	7.8	2.2
West Virginia	2	16	3	81%	19%	1.8	9
Wisconsin	2	25	0	100%	0%	5.9	4.2

*NRMP*, National Resident Matching Program.

### Hospital Ownership

The majority (63%) of residency EM positions were offered by 177 programs at non-profit hospitals (1,880/3,010), while 68 public hospital programs offered 28% of positions (831/3,010), and 31 for-profit hospital programs offered 10% of positions (299/3,010). There was a statistically significant difference in the percentage of unmatched positions by hospital ownership type (*P* < 0.001) (Table 2). Public hospitals had a statistically significant higher match rate (88%), compared to non-profit and for-profit hospitals, which had match rates of 80% and 75%, respectively (*P* < 0.001). There was no difference in match rates between non-profit and for-profit hospitals. Seventeen health systems operated three or more residency programs, of which 11 were non-profit, three were for-profit, and two were public. The health system offering the largest number of residency programs was HCA Healthcare (19 programs, 189 positions, 70% match rate).

**Table 2.** Association of hospital ownership type on unfilled emergency medicine positions.

Health system	Ownership type	Number of residency programs	NRMP positions available	NRMP positions matched	Unmatched positions (%)
By hospital ownership type (*P* < 0.001, Pearson chi-squared test)					
	For profit	31	299	224	25.1%
	Non-profit	177	1880	1502	20.1%
	Public	68	831	730	12.2%
	Total	276	3010	2456	18.4%
By health system/type (operating 3+ EM residencies)					
Ascension Health	Non-profit	7	64	42	34.4%
Baylor Scott & White Health	Non-profit	3	28	23	17.9%
Bon Secours Mercy Health	Non-profit	3	28	15	46.4%
Corewell Health	Non-profit	5	50	36	28.0%
HCA Healthcare	For profit	19	189	132	30.2%
Henry Ford Health System	Non-profit	4	40	18	55.0%
Jefferson Health	Non-profit	5	59	42	28.8%
Michigan Medicine	Public	3	30	23	23.3%
NewYork-Presbyterian	Non-profit	3	43	42	2.3%
Northwell Health	Non-profit	3	39	34	12.8%
NYC Health + Hospitals	Public	6	85	72	15.3%
RWJ Barnabas Health	Non-profit	3	29	23	20.7%
Tenet Healthcare	For profit	4	44	40	9.1%
Trinity Health	Non-profit	6	41	18	56.1%
Universal Health Services	For-profit	3	30	24	20.0%
University of California	Public	5	67	67	0.0%
UPMC	Non-profit	3	28	24	14.3%
	Total	85	894	675	24.5%

Overall, the proportions of filled/unfilled positions did vary by hospital ownership type (X2 = 34.126, df = 2, *P* < 0.001). Post-hoc Bonferroni comparisons between hospital types showed that public hospitals had a lower proportion of unfilled positions compared to both for-profit and non-profit hospitals (raw and adjusted *P*-values <0.001), while there was no difference in the proportion of positions filled between for-profit and non-profit hospitals (raw *P* = 0.05, adjusted *P* = 0.16).

NRMP = National Resident Matching Program.

*UPMC*, University of Pittsburgh Medical Center.

### Group Ownership and Employment Model

Among EM faculty group ownership and employment models, half of EM residency positions (52%) had program faculty that were employed by health systems (1,574/3,010, 134 programs), with 31% having clinician partnership faculty (941/3,010, 87 programs), and 16% of positions having private equity-employed faculty (495/3,010, 55 programs). Five employer groups met the definition of majority private equity ownership. These groups included American Physician Partners, Envision Physician Services, SCP Health, Sound Physicians, and TeamHealth.

There was a statistically significant difference in the percentage of unmatched positions by the employment model of faculty physicians (*P* < 0.001) (Table 3). Programs with faculty employed by a health system had the highest match rate of 87%, followed by clinician partnerships at 79% and private equity groups at 68% (Table 3). Thirteen physician groups operated three or more residency programs. The physician groups staffing the largest number of residency programs were Envision Physicians Services (24 programs, 230 positions, 71% match rate) and TeamHealth (21 programs, 197 positions, 75% match rate).

**Table 3.** 2023 emergency medicine match rates by faculty physician group/type.

Physician group	Group type	Number of residency programs	NRMP positions available	NRMP positions matched	Unmatched positions (%)
By residency faculty physician group type (*P* < 0.001, Pearson chi-squared test)					
Health system (HS)		134	1574	1375	13%
Clinician partnership (CP)		87	941	744	21%
Private equity (PE)		55	495	337	32%
	Total	276	3010	2456	18.4%
By residency faculty group (operating 3+ EM residencies)					
American Physician Partners	PE	4	26	6	77%
ApolloMD	CP	4	36	23	36%
Envision Physician Services	PE	24	230	163	29%
Integrative Emergency Services	CP	3	29	24	17%
Northwell Health	HS	3	39	34	13%
Physician Affiliate Group of New York	CP	7	98	84	14%
RWJ Barnabas Health	HS	3	29	23	21%
SCP Health	PE	4	28	14	50%
TeamHealth	PE	22	205	150	27%
University of California	CP	5	67	67	0%
UPMC	HS	3	28	24	14%
US Acute Care Solutions	CP	7	57	28	51%
Vituity	CP	11	115	89	23%
	Total	100	987	729	26%

Overall, the proportions of filled/unfilled positions did vary by residency faculty physician group type (X2 = 99.007, df = 2, *P* < 0.001). Post-hoc Bonferroni comparisons between group types showed that programs with health system employed faculty had the lowest proportion of unfilled positions, followed by clinician partnership faculty, while residencies with private equity employed faculty had the highest proportion of unfilled positions (raw and adjusted p-values for all pairwise comparisons <0.001).

*NRMP*, National Resident Matching Program; *UPMC*, University of Pittsburgh Medical Center.

### Program and Hospital-specific Attributes

When comparing filled and unfilled programs by accreditation history and hospital-level characteristics, unfilled programs were more likely to be smaller in size based on the number of positions offered (*P* < 0.001), previously accredited by the AOA (*P* < 0.001), and started in more recent years (*P* < 0.001). There was no difference in filled vs unfilled programs by program length (*P* = 0.78). Unfilled programs tended to be in less urban areas (*P* = 0.03), at hospitals with a smaller number of beds (*P* < 0.001), lower resident-to-bed ratios (*P* < 0.001), and fewer disproportionate share patients (*P* < 0.001) (Table 4).

**Table 4.** Comparing attributes of filled/unfilled programs in 2023 emergency medicine match.

	Filled (n = 145)	Unfilled (n = 131)	Total	*P*-value
Original accreditation type				<0.001^a^
ACGME	141 (97%)	84 (64%)	225 (82%)	
AOA	4 (3%)	47 (36%)	51 (19%)	
Year of original accreditation				<0.001^b^
Median	1995	2010	2003	
Q1, Q3	1982, 2009	1993, 2018	1988, 2016	
Year of ACGME accreditation				<0.001^b^
Median	1995	2017	2008	
Q1, Q3	1982, 2011	2006, 2019	1990, 2017	
Total approved ACGME positions				<0.001^b^
Median	39	30	36	
Q1, Q3	30, 54	22, 36	24, 44	
Length of training				0.78^a^
3 years	116 (80%)	103 (79%)	219 (80%)	
4 years	29 (20%)	28 (21%)	57 (21%)	
Urban-rural				0.03^a^
Large urban area	89 (61%)	64 (49%)	153 (55%)	
Other urban area	55 (38%)	61 (47%)	116 (42%)	
Rural area	1 (1%)	6 (5%)	7 (3%)	
Number of hospital beds				<0.001^b^
Median	571	359	450	
Q1, Q3	382, 730	260, 534	318, 680	
Resident-to-bed ratio (per 100 beds)				<0.001^b^
Median	47	29	38	
Q1, Q3	30, 70	16, 45	21, 63	
Disproportionate share hospital patients [%]				<0.001^b^
Median	39	33	36	
Q1, Q3	31, 52	28, 43	30, 47	

^a^ Pearson chi-squared test.

^b^ Wilcoxon rank-sum test.

*ACGME*, Accreditation Council for Graduate Medical Education; *AOA*, American Osteopathic Association; *Q*, quartertile.

## DISCUSSION

We examine the factors and program characteristics associated with unfilled positions in the EM match. Five states were associated with two-thirds of the unfilled positions. Public hospitals had a statistically significant higher match rate (88%) when compared to non-profit and for-profit hospitals, which had match rates of 80% and 75%, respectively (*P* < 0.001). Public hospitals include those owned by government entities (local, state, federal government) or the Veterans Health Administration. Non-profit and for-profit hospitals are privately owned and differentiated by their tax status (discussed further below). Programs with faculty employed by a health system had the highest match rate of 87%, followed by clinician partnerships at 79% and private equity groups at 68% (*P* < 0.001 overall and between all subgroups). Our analysis confirms and expands findings from recent studies. One study identified six characteristics of unfilled programs (in descending order of predictive strength): unfilled positions in the 2022 match; smaller program size; Mid-Atlantic location; prior AOA accreditation; East North Central location; and private equity majority ownership of physician faculty group.^3^ Another study of combined 2022 and 2023 match data found programs at risk of not filling had accreditation within the prior five years, had a for-profit primary clinical site, and were in geographic areas with high numbers of positions offered.^2^

### Residency Growth Trends

The number of unmatched positions in the EM match was driven by a dramatic increase in the number of EM programs and positions offered over the past decade, as well as a more recent decrease in applicants over the prior two years. Between 2014–2023, there was a 29% increase in the number of EM programs and a 46% increase in the number of postgraduate year (PGY)-1 positions offered in the match, suggesting that the growth of positions is not only related to the creation of new programs but also the expansion of existing programs. In recent years, EM has experienced the largest growth rate of PGY-1 positions across all medical specialties.^7^ The match rate is also impacted by a decrease in the number of applicants over time. Applicants in EM peaked in 2021 at 4,391 applicants. It is unclear whether this record high, representing a 16% increase over the year before, was an outlier. The overall five-year trend is an 8% decrease in applicants contrasted with the 23% increase in positions.^8^ This unprecedented growth has outstripped the number students applying to train in EM and played a large role in the number of unfilled spots in 2023.

Between 2013–2020, there was significant growth of EM residencies in states that already had multiple EM training programs. A number of states nearly doubled the number of training programs in that time frame: New York (21 to 31), Pennsylvania (12 to 21), and California (14 to 22), while others grew even more Ohio (9 to 18), Michigan (11 to 25), and Florida (5 to 19).^9^^,^^10^ New programs are disproportionately growing in urban areas, whereas some rural states do not have any EM training programs.^10^ Only seven EM residency programs are located in rural areas, six of which did not fill.^11^ Our data demonstrates that many of the unfilled spots in 2023 occurred in states that had the highest absolute number of resident positions as well as number of residents per capita population. No state-level regulations exist to limit the number of residency training programs. While some have called on the ACGME to restrict the number of EM training positions, it is currently against ACGME policy and a violation of state and federal antitrust law for the ACGME to implement a national workforce policy to establish the number of practicing physicians.^12^ The ACGME can create and adjust standards for accreditation to optimize the learning environment. Some have expressed concern regarding the academic quality of some of the newer programs. One study found that nearly 25% of programs were given “with warning” accreditation on initial accreditation compared to less than 3% of programs on continued accreditation.^7^^,^^13^

Debate exists over who is responsible for the increased growth of residency programs. A new residency program requires a sponsoring institution, which the ACGME defines as an “organization or entity that assumes ultimate financial and academic responsibility for a program.” Sponsoring institutions may include universities, medical schools, hospitals, healthcare delivery systems, or physician group practices.^14^ Currently, a review of the ACGME listings reveals that all EM residency programs are sponsored by hospitals and health systems, with none being sponsored by physician staffing groups.^13^ The role and motivation of the physician groups who serve as faculty for new residency programs that are sponsored by hospitals and health systems may vary. Graduate medical training programs offer financial benefits to hospitals and recruitment benefits to hosting institutions and staffing groups.^15^ New program growth could be driven at the physician group, hospital or health system level, or both. For example, HCA Healthcare has a transparent objective to expand GME positions stating, “With 270+ residency and fellowship programs, HCA Healthcare plans to continue to grow the largest GME community in the United States.”^16^ It is reasonable to surmise that faculty groups feel pressure to start and staff new programs to align with the health system’s intent to maintain contracts. Hospitals that created GME programs after 2015, known as “GME-naive,” have a strong incentive to increase the number of residents at their site within five years of starting because CMS calculates their training cap after the fifth year.^17^

Unfilled spots may represent market forces rightsizing the number and geographic distribution of residency slots, although the complexities of GME funding and training caps create regulatory barriers to market corrections.^9^ Unfilled positions do not receive GME funding, which could lead to residency closures without alternate sources of funding.^18^ When anesthesia experienced a similar plight of decreasing fill rates in the 1990s, a cumulative drop of 77% of applicants over a six-year period resulted in 16% of all anesthesia residencies in the country closing their doors.^11^ However, market corrections will not occur if unfilled spots in the initial match are subsequently filled in the SOAP, which occurs a few days later. Most of the unfilled EM positions in the 2022 Match subsequently filled in the SOAP.^19^ Discussion continues on how best to maintain the quality and stability of the EM workforce.^1^

### Corporations and Graduate Medical Education

We observed significant differences in match rates by hospital ownership type with public hospitals having the fewest unmatched positions. Non-profit hospitals continue to make up the majority of EM training sites, and there was no statistical difference in match rates between non-profit and for-profit hospitals. Over the past 20 years, there has been increased consolidation and corporatization in healthcare including EM practice and training.^20^^–^^22^ Many fear that increased for-profit and investor sponsorship of residency programs may result in lower quality training or the commoditization of GME.^23^^,^^24^ While there has been increased scrutiny on corporate investment in healthcare and medical education, and some studies on health or workforce outcomes in other specialties, no such studies exist in EM.^20^^,^^25^^,^^26^ The proportion of EM residencies created at for-profit hospitals has increased considerably.^7^ Prior to 2016, only 5% of EM residency programs had primary sites at for-profit hospitals (10 total), compared to 30% (21/71) of new programs being based at for-profit hospitals. While hospitals are frequently differentiated by non-profit or for-profit status, this differentiation based on tax status has limitations in capturing the business incentives of the institution.^27^

Our data shows that public hospitals were associated with the highest match rates. There was no difference between for-profit and non-profit hospitals with regard to match rates. A prior study similarly did not find a statistically significant different greater risk of not filling at for-profit sites (compared to non-profit or government sites) but did find a 50% greater risk of not filling when examining 2022 and 2023 match data.^2^ We did find significant variation between groups within the same tax designation. For example, of the 17 health systems that operate three or more programs, Trinity Health, a non-profit health system, had the highest percentage of unmatched positions at 56% (six programs total, 23/41 unmatched) and the University of California, a public health system, had 0 unfilled positions (five programs total, 67 positions). The health system operating the largest number of EM residencies is HCA Health, a for-profit health system, which offered 189 positions at 19 programs, of which 30% were unmatched. Tenet Healthcare, another for-profit health system, which offered 44 positions at four programs, had a match rate above the national average, filling 91% of its positions. Hence, although public hospitals had a higher match rate overall, there is significant variability.

Much scrutiny has focused on corporate, specifically private equity (PE), ownership and investment in EM. Among the different types of non-physician corporate investors, PE has undergone particular criticism due to significant expansion within EM, evidence of poor outcomes in other areas of healthcare, and short-term profit incentives.^28^ Private equity and publicly traded company control of the emergency physician staffing market increased from 8.6% to 22% from 2009 to 2019.^26^ Private equity-acquired hospitals now account for 8% of all nongovernmental hospitals.^29^ Our data shows that 503/3,010 (17%) of EM residency positions in the 2023 match were staffed by physician groups that are majority owned by PE. To our knowledge, there has never been an outcomes comparison study between employment models within residency training programs to predict success in practice after graduation. Employment models of physicians are changing with increased consolidation in healthcare. Emergency medicine-bound students have expressed concern about corporate influence in EM, but it is unclear the relative contribution of this on student recruitment especially in light of other factors.^30^ Academic faculty can be employed in multiple employment models such as by a medical school, a health system, a large national group, a regional group, or a single ownership group. Emergency medicine programs with the highest fill rates in the match were associated with employment models in which faculty were directly employed by the hospital, health system, or medical school. There was significant variability, however, between employers and employment types.

## LIMITATIONS

This analysis has several important limitations. There are many reasons a medical student may rank and matriculate at a residency program. Unique characteristics of a program that may influence a particular applicant’s interest and rank list were not captured for analysis. The number of applicants interviewed and ranked by programs are additional factors that impact match rates, which were not measured. The past two years did not include in-person applicant interviews, which may have also impacted match rates.

Additionally, the relationships between hospitals, health systems, physician faculty groups, and individual residency programs are complex and evolving, and this must be considered when interpreting results. For example, one health system may employ physicians under multiple models such as direct employment or a third-party staffing group. The current health system or staffing group at the program in this analysis may not have been the same one present when the residency started due to mergers and acquisitions. Since this analysis there have been major changes in the emergency physician staffing landscape including the closure of American Physicians Partners and Chapter 11 Bankruptcy of Envision, which operated four and 24 residencies in the 2023 EM match, respectively.^31^

There are no currently agreed upon definitions for classifying physician-group ownership structures. The varied spectrum of corporate investor (eg, PE) ownership stakes in EM groups from minority to whole complicates the creation of discrete categories. Our classification of health systems was not able to differentiate between the various complex relationships that comprise health systems, such as as whether the health system physician group is wholly owned by the health system and or they are owned by a medical school, academic medical center, or hospital. Most fundamentally, ownership only serves as a proxy for other important features such as physician autonomy and educational quality.

## CONCLUSION

The 2023 match in EM saw increased rates in the number of training slots and programs that did not fill before the SOAP. Public hospitals had higher match rates than for-profit or non-profit hospitals overall, but there was significant variability within hospitals and health systems. Residency programs that employed academic faculty directly through the hospital or health system were associated with higher match rates.
